# A New Sesquiterpenoid Aminoquinone from an Indonesian Marine Sponge

**DOI:** 10.3390/md17030158

**Published:** 2019-03-08

**Authors:** Walter Balansa, Ute Mettal, Zerlina G. Wuisan, Anuchit Plubrukarn, Frans G. Ijong, Yang Liu, Till F. Schäberle

**Affiliations:** 1 Institute for Insect Biotechnology, Justus-Liebig-University of Giessen, 35392 Giessen, Germany; walterbalansa1@gmail.com (W.B.); Ute.Mettal@chemie.uni-giessen.de (U.M.); Zerlina.G.Wuisan@bio.uni-giessen.de (Z.G.W.); 2 Department of Fisheries and Marine Science, Nusa Utara Polytechnic, Tahuna 95812, North Sulawesi, Indonesia; ijongfrans@yahoo.com; 3 Department of Bioresources of the Fraunhofer Institute for Molecular Biology and Applied Ecology, 35394 Giessen, Germany; 4 Department of Pharmacognosy and Pharmaceutical Botany, Faculty of Pharmaceutical Sciences, Prince of Songkla University, Songkhla 90110, Thailand; anuchit.pl@psu.ac.th; 5 Faculty of Fisheries and Marine Science, Sam Ratulangi University, Manado 95115, Indonesia; 6 German Center for Infection Research (DZIF), Partner Site Giessen-Marburg-Langen, 35392 Giessen, Germany

**Keywords:** nakijiquinone, antibacterial activity, cytotoxicity, merosesquiterpenes, aminoquinone

## Abstract

Sponges are a well-known bioresource for bioactive compounds. In this study, antibacterial activity-guided fractionation of the extract from an Indonesian marine *Dactylospongia elegans* sponge led to the discovery of four merosesquiterpenoids, namely, a new sesquiterpenoid aminoquinone nakijiquinone V (**1**), along with illimaquinone (**2**), smenospongine (**3**), and dyctioceratine C (**4**). The structure of compound **1** was elucidated by 1D and 2D NMR as well as by LC-HRESIMS data analysis. Compounds **2**–**4** showed moderate to low antimicrobial activity against *Bacillus megaterium* DSM32 with a minimum inhibitory concentration (MIC) of 32 μg/mL, 32 μg/mL, and 64 μg/mL, respectively. Furthermore, compounds **2** and **3** both inhibited *Micrococcus luteus* ATCC 4698 with a MIC of 32 μg/mL. In conclusion, the isolated merosesquiterpenoids, which are known for their cytotoxic effects, showed antibacterial activity and prompt future structure activity relationship (SAR) studies concerning the various bioactivities observed for this group of natural products.

## 1. Introduction

Sponges are a prolific source of bioactive compounds [1,2,3,4,5,6,7]; in particular, the genera *Dysidea*, *Spongia*, and *Dactylospongia* (family Spongidae) represent a rich source of bioactive sesquiterpenoid quinones and hydroquinones [8]. Merosesquiterpenes contain a sesquiterpene unit joined to a phenolic or quinone moiety and arise from a mixed polyketide-terpenoid biosynthetic pathway. [9,10] These merosesquiterpenoids continue to attract considerable attention due to their structural diversity and intrinsic biological activities [11] including, but not limited to, antimicrobial [8,12], anti-HIV [13,14], Golgi disruptor agents [15], potent hypoxic inducers in prostate cancer cell lines [16,17], and apoptotic inducers in leukemic cells [18]. Over the years, more than 70 sesquiterpene quinones/hydroquinones have been described in the literature, mainly featuring drimane or rearranged drimane skeletons [19].

**Figure 1:**
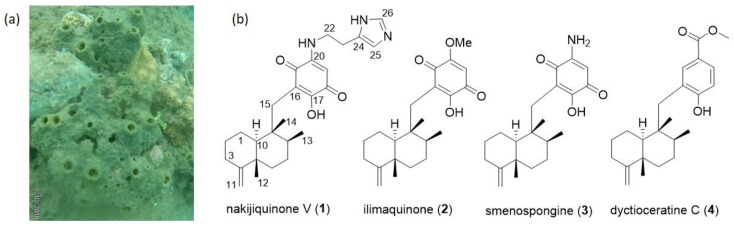
(**a**) Underwater picture of the sponge *Dactylospongia elegans* T3; (**b**) Structures of the isolated compounds **1**–**4**.

During our ongoing search for new antibiotic compounds from Indonesian marine sponges, we investigated the extract of a sponge specimen, identified as *Dactylospongia elegans* based on 28S rRNA gene barcoding, which was collected from Tahuna, Sangihe Islands (Figure 1a). The extract showed antimicrobial activity against *Bacillus megaterium* DSM32 and *Micrococcus luteus* ATCC 4698. The bioactivity encouraged us to further investigate the chemical diversity of the bioactive extract. Herein, we report on the isolation, structure elucidation, and biological activity of the secondary metabolites from this Indonesian marine sponge.

## 2. Results

When the extract was subjected to HPLC analysis, it showed the characteristic UV absorption pattern of the sesquiterpene quinone/hydroquinone system (Figure S8). Detailed chemical investigation of the extract resulted in the isolation of one new sesquiterpene aminoquinone (**1**), two known sesquiterpene quinones (**2–3**), and one known sesquiterpene hydroquinone (**4**). Based on the obtained NMR and MS data, a comparison with the literature led to the identification of the known compounds (**2**–**4**), illimaquinone (**2**) [20], smenospongine (**3**) [21], and dyctioceratine C (**4**) [22] (Figure 1b).

Compound **1** was obtained as a purple amorphous solid with an optical rotation value of ${\left[\alpha \right]}_{D}^{24}=+54$ (*c* 0.08, MeOH). Its molecular formula was established as C_26_H_35_N_3_O_3_ based on the prominent pseudomolecular ion peaks at *m/z* 438.2764 [M + H]^+^ and 460.2575 [M + Na]^+^ in the LC-HRESIMS spectrum (Figure S7). The ^13^C NMR spectrum (Table 1, Supplementary Figure S2) showed one signal for the carbonyl group, nine olefinic/aromatic carbons—three of which were methine and one was an *exo*-methylene—three methyl groups, eight aliphatic methylenes, two aliphatic methines, and two aliphatic quaternary carbons.

**Figure 2:**
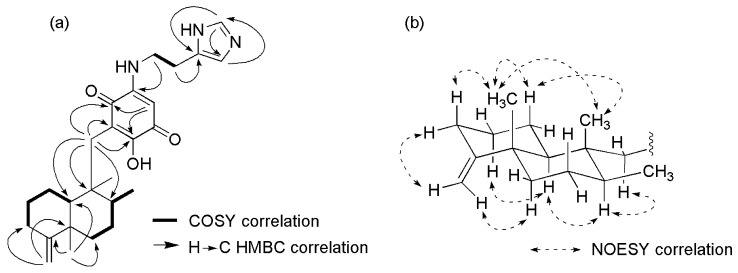
(**a**) Selected COSY, HMBC correlations for compound **1**; (**b**) Selected NOESY correlations for compound **1**.

The ^1^H NMR spectrum of **1** (Table 1, Supplementary Figure S1), particularly in the aliphatic region, resembled that of compounds **2**–**4**, suggesting that all possessed a similar sesquiterpene skeleton. Analysis of the ^1^H NMR, HSQC (Supplementary Figure S4) and COSY (Supplementary Figure S3) spectra allowed for the assembly of two spin systems; one from H-10 through H-1 and H-2 to H-3, and the other from H-6 through H-7 and H-8 to H-13 (Figure 2a). Connection of the two spin systems was done through the HMBC correlations from H-11 (*δ* 4.42) to C-3 (*δ* 34.1) and C-5 (*δ* 41.6); from H-12 (*δ* 1.05) to C-4 (*δ* 161.7), C-5, C-6 (*δ* 38.1), and C-10 (*δ* 51.3); and from H-15 (*δ* 2.49 and 2.39) to C-8 (*δ* 39.1), C-9 (*δ* 43.9), and C-10 (Figure 2a, Supplementary Figure S5). Hence, a friedodrimane-type sesquiterpene skeleton functionalized by a 4,11-exo-methylene moiety was furnished. In the downfield region of the ^1^H NMR spectrum, two aromatic protons at *δ* 8.77 and 7.35 (H-26 and H-25) were observed, which were thoroughly connected through HMBC correlations (Figure 2a) with three carbons at *δ* 135.1, 132.5, and 117.8 (C-26, C-24, and C-25), thus forming a spin system, characteristic of an imidazole moiety. Placement of the carbons, C-21 (*δ* 184.1), C-20 (*δ* 151.8), and C-19 (*δ* 93.0) onto the quinone moiety were based on their characteristic chemicals shifts, and were supported by the HMBC correlations from H-19 (*δ* 5.38) to C-17 and C-21. The sole hydroxy group was attached to C-17 (*δ* 159.6) based on the low-field ^13^C chemical shift. According to the degree of unsaturation (unsaturation index = 11) indicated by the molecular formula, there should be one more carbonyl group (C-18, *δ* 179.1), which only gave a very low intensity resonance signal in the ^13^C NMR spectrum to establish the quinone moiety. This quinone moiety is connected to the aforementioned imidazole over an amino ethylene bridge (*δ* 3.54 and 3.05; H-22 and H-23; and *δ* 42.2 and 24.3; C-22 and C-23). The resulting histaminyl unit was connected to C-20 as indicated by the HMBC correlations from H-22 to C-20 and from H-23 to C-24, and supported by the dipolar couplings between the ethyl protons and H-19 of the quinone. The remaining HMBC correlations from H-15 to C-16 (*δ* 115.7), C-17 (*δ* 159.6), and C-21 (*δ* 184.1) further allowed for the connection between the friedodrimane and quinone moieties via the methylene bridge C-15. Comparison of the NMR data of compound **1** to those of nakijiquinone G [23] showed that the two compounds were nearly identical except that compound **1** had three methyl groups attached to a decalin system with an exocyclic instead of the endocyclic double bond in contrast to its counterpart nakijiquinone G [23]. Hence, compound **1** was identified as a new natural sesquiterpenoid aminoquinone for which the name nakijiquinone V was proposed.

The relative configuration of **1** was determined on the basis of the signal profile and NOESY analyses (Supplementary Figure S6). The characteristic chemical shifts of C-12 (*δ* 21.0), C-14 (*δ* 17.8), and C-4 (*δ* 161.7) and the coupling constant of H-10 (11.8 Hz) indicated that all three occupied the axial orientation, hence a *trans*-decalin system for the friedodrimane core skeleton [24], similar to the relative configuration of **2**–**4**, was assigned. This was supported by the NOESY experiment, from which the dipolar couplings between H-12 and H-14, and among H-8, H-10, and H-15b were observed, indicating that the two methyl substituents, H-12 and H-14, resided on the same plane, whereas H-8, H-10, and all of the quinone moiety extensions were on the opposite (Figure 2b). The axial and equatorial orientation of all of the alicyclic methylene protons, except for H-7, was also assigned according to their coupling constants, and were confirmatively supported by the NOESY spectral analyses (Figure 2b).

Compounds **1**–**4** were investigated for their antibacterial activity toward two Gram-positive bacteria (*M. luteus* ATCC 4698, *B. megaterium DSM32*) and one Gram-negative bacterium (E. coli K12 derivative.) Although all compounds lacked antimicrobial activity against E. coli, compounds **2–4** exhibited modest antimicrobial activity against *B. megaterium* with MIC values of 32 μg/mL, 32 μg/mL, and 64 μg/mL, respectively, and **2**–**3** against *M. luteus* with a MIC of 32 μg/mL each (Supplementary Figure S9).

The 28S rRNA sequence of the sponge (GenBank accession number: MK554863) showed the highest homology to the *Dactylospongia elegans* strain SCS1. Therefore, the here reported sponge was named *Dactylospongia elegans* T3.

## 3. Materials and Methods

### 3.1. General Experimental Procedures

Optical rotation was measured on a Jasco P-2000 polarimeter (JASCO Deutschland GmbH, Pfungstadt, Germany). The λ_max_ of compound **1** was measured on a Jasco V760 spectrometer spectrometer (JASCO Deutschland GmbH, Pfungstadt, Germany). NMR spectra were recorded in CD_3_OD (ALDRICH, St. Louis, MO, USA) using Bruker Avance II 400 MHz and Brucker Avance III HD 600 MHz NMR spectrometers for known and new compounds, respectively (all: Brucker, Ettlingen, Germany). Analysis of NMR spectra was done using the software MestReNova 10 (MESTRELAB RESEARCH SL, Santiago de Compostela, Spain). Mass spectra were recorded on a micrOTOF-Q mass spectrometer (Bruker, Billerica, MA, USA) with an ESI-source coupled with a HPLC Dionex Ultimate 3000 (Thermo Scientific, Darmstadt, Germany) using an EC10/2 Nucleoshell C18 2.7 µm column (Macherey-Nagel, Düren, Germany). Spectral analysis was done using Bruker Data Analysis (Bruker, Billerica, MA, USA). The column temperature was 25 °C. MS data were acquired over a range from 100 to 1000 *m/z* in positive mode. Auto MS/MS fragmentation was achieved with rising collision energy (35–50 keV over a gradient from 500 to 2000 *m/z*) with a frequency of 4 Hz for all of the ions over a threshold of 100. The injection volume was 2 µL with a concentration of 1 mg/mL. Fractionation was performed on the Interchim Puriflash 4125 chromatography system (Interchim, Montluçon, France) and purification on the Shimadzu HPLC (Shimadzu Deutschland GmbH, Duisburg, Germany).

### 3.2. Sponge Material

The sponge specimen *Dactylospongia elegans* T3 was collected by hand using SCUBA from Towo’e Beach Tahuna Bay, Sangihe Islands North Sulawesi Province Indonesia at the depth of ~4 m in June 2018. The sponge is greenish gray (olive) underwater but turns to a pale sandy yellowish-brown (beige) when exposed to air. The shape of the colony was amorphous with the size of 31.3 × 8.2 cm^2^ and the oscular of 2–3 cm in diameter. The pungent garlic odor and soft texture sponge produced slime when touched or cut with the inner part of the sponge being plum. The voucher specimen is preserved at the Institute of Insect Biotechnology Justus Liebig University of Giessen, Germany.

### 3.3. Extraction and Isolation

The sponge (300 g wet weight) was cut into small pieces and dried in the oven at 45 °C for two days prior to transport to Germany. The dried specimen (49.3 g dried weight) was soaked in 400 mL MeOH (gradient grade for HPLC, Chemsolute, Th. Geyer GmbH & Co. KG, Renningen, Germany) overnight (2×), filtered and the combined supernatants were dried under reduced pressure to yield 4.1 g crude extract. It was fractionated by a reverse phase flash chromatography (Interchim Puriflash 4125 chromatography system equipped with a Puriflash C18-HP30 mm Flash column, gradient elution of 5% MeOH/H_2_O to 100% MeOH over 1 h) to give 17 fractions. Fraction 14 (125.0 mg) was further purified by HPLC (semi preparative column, EC Gravity C_18_, 250 × 10 mm, gradient elution, 88% MeOH/H_2_O to 95% MeOH/H_2_O + 0.01% TFA, flow rate 3.0 mL/min., over 30 min.) to give four subfractions, of which the major subfractions 2 and 4 were already pure and identified as illimaquinone (**2**) (15.0 mg) and dyctioceratine C (**4**) (5.0 mg), respectively. Further purification by HPLC of subfraction 1 (EC Gravity C_18_, 250 × 10 mm, 77%MeOH/H_2_O isocratic + 0.01% TFA over 30 min.) and of subfraction 3 (EC Gravity C_18_, 250 × 10 mm, 80%MeOH isocratic + 0.01% TFA over 30 min.) resulted in nakijiquinone V (**1**) (1.9 mg) and smenospongine (**3**) (2.3 mg), respectively.

Nakijiquinone V (**1**): Purple amorphous solid; ${\left[\alpha \right]}_{D}^{24}=+54$ (*c* 0.08, MeOH); UV [MeOH; *λ*_max_ in nm]: *λ*_max_ (log *ε*) 308 (5,08); ^1^H (600 HMz) and ^13^C (150 MHz) NMR (CD_3_OD), see Table 1; LC-HRESIMS *m/z* 438.2764 [M + H]^+^ and 460.2575 [M + Na]^+^ (calculated for C_26_H_36_N_3_O_3_, 438.2751).

### 3.4. Antimicrobial Assay

Antimicrobial-guided isolation was performed based on the disk diffusion method. LB agar plates (10 g peptone, 5 g yeast extract, 5 g NaCl, 15 g agar, mixed with 1 L distilled water) were prepared and the respective test bacteria (*Bacillus megaterium* DSM32, *Micrococcus luteus* ATCC4698, *Escherichia coli* K12) were spread onto it. A total of 15 µL of a methanol solution of the compound (10 mg/mL) were added to a paper disk, methanol was used as the negative control and carbenicillin (5 µL of a 50 mg/mL stock solution) (Carl Roth GmbH + Co., Karlsruhe, Germany) was used as positive control, which was subsequently positioned on the agar plate after drying. Incubation was performed at 37 °C overnight.

The minimum inhibitory concentration (MIC) was determined in a liquid medium. Therefore, compounds **2**–**4** were dissolved in dimethyl sulfoxide (DMSO, Carl Roth GmbH + Co., Karlsruhe, Germany) and diluted to the following final concentrations: 128, 64, 32, 16, 8, 4, 2, 1, 0.5, and 0.25 μg/mL. The compound was added into each well of the 96-well plate, filled previously with a *Bacillus megaterium* DSM32, *Micrococcus luteus* ATCC4698, or *Escherichia coli* K12 solution with an optical density (OD_600_) of 0.1 in Luria Bertani broth (final volume: 200 μL per well). Pure DMSO was used as the negative control, and carbenicillin as the positive control (2 µg/mL). The OD_600_ was immediately measured as the initial optical density value. The plates were incubated at 30 °C with 160 rpm for 24 h. Then, the OD_600_ was measured to check the culture growth in the wells in comparison to the positive control, i.e., the OD_600_ value of the initial measurement was subtracted from the 24 h value, and divided by the OD_600_ value of the negative control.

### 3.5. Sponge Identification

A small piece of the sponge specimen was added into 400 µL lysis mix (100 mM NaCl, 50 mM Tris-HCl pH 8.0, 10 mM EDTA pH 8.0, 0.5% SDS, 2 mg/mL Proteinase K) and incubated at 56 °C overnight [25]. Thereafter, the genomic DNA was isolated using the innuPREP Bacteria DNA Kit (Analytik Jena, Jena, Germany) and dialyzed using a 0.025 µm nitrocellulose membrane (MerckkGaA, Darmstadt, Germany). Prior to PCR, the sponge genomic DNA was diluted 1:10.

The following barcoding primers were used [26]: C2: 5′-GAAAAGAACTTTGRARAGAGAGT-3′ and D2: 5′-TCCGTGTTTCAAGACGGG-3′ to amplify the 28S rRNA fragment from the isolated sponge. Q5^®^ High-Fidelity DNA Polymerase (New England Biolabs, Ipswich, MA, USA) was used for PCR using the following program: initial denaturation at 95 °C for 2 min; 34 cycles of 95 °C for 45 s, 56 °C for 45 s, 72 °C for 45 s; final elongation at 72 °C for 5 min.

The 28S rRNA amplificate was sent to Microsynth Seqlab, Göttingen, Germany, for Sanger sequencing. Sequence reads were assembled in Clone Manager 9 (Scientific & Educational Software, Denver, CO, USA) and aligned by BLAST (NCBI) to determine the sponge identity.

## 4. Conclusions

In conclusion, by applying bioassay-guided fractionation, four merosesquiterpenes were obtained from an Indonesian marine *Dactylospongia elegans* sponge. Compounds **2**–**4** exhibited modest antimicrobial activity against Gram-positive test strains. However, the class of isolated compounds, which is known for their cytotoxic effects, deserves future SAR-studies to obtain more insights into their potential as anti-cancer molecules.

## Figures and Tables

**Table 1:** ^1^H (600 MHz) and ^13^C (150 MHz) NMR data of **1** (CD_3_OD; *δ* in ppm).

Position	*δ*_H_, Mult. (*J* in Hz)	*δ*_C_, Type	Position	*δ*_H_, Mult. (*J* in Hz)	*δ*_C_, Type
1ax	1.45, m	24.3, CH_2_	13	0.98, d (6.4)	18.5, CH_3_
eq	2.15, br d (14.0)		14	0.84, s	17.8, CH_3_
2ax	1.17, m	29.9, CH_2_	15a	2.49, d (13.7)	33.2, CH_2_
eq	1.82, dd (12.7, 2.7)		b	2.39, d (13.7)	
3ax	2.34, ddd (13.9, 13.5, 5.3)	34.1, CH_2_	16	-	115.7, C
eq	2.04, br dd, (13.5, 4.5)		17	-	159.6, C
4	-	161.7, C	18	-	179.1^,^ C
5	-	41.6, C	19	5.38, s	93.0, CH
6ax	1.30, ddd (12.7, 12.4, 3.7)	38.1, CH_2_	20	-	151.8, C
eq	1.51, br d (12.4)		21	-	184.1, C
7	1.38, m (2H)	29.1, CH_2_	22	3.54, t (6.9)	42.2, CH_2_
8	1.21, m	39.1, CH	23	3.05, t (6.9)	24.3, CH_2_
9	-	43.9, C	24	-	132.5, C
10	0.78, dd (11.8, 1.5)	51.3, CH	25	7.35, s	117.8, CH
11	4.42, s (2H)	103.6, CH_2_	26	8.77, s	135.1, CH
12	1.05, s (3H)	21.0, CH_3_			

## Supplementary Materials

NMR spectra for compound **1** are available online at https://www.mdpi.com/1660-3397/17/3/158/s1, Figures S1–S6: NMR data of compound **1**. Figure S7: LC-HRESIMS of compound **1**. Figure S8: UV spectra of compounds **1**–**4**. Figure S9: Diagrams for MIC determination of compounds **2**–**4**.

## References

[1] Yamazaki H., Wewengkang D.S., Kanno S., Ishikawa M., Rotinsulu H., Mangindaan R.E., Namikoshi M. (2013). Papuamine and haliclonadiamine, obtained from an Indonesian sponge *Haliclona* sp., inhibited cell proliferation of human cancer cell lines. Nat. Prod. Res..

[2] Salmoun M., Braekman J.C., Dewelle J., Darro F., Kiss R., De Voogd N.J., Van Soest R.W.M. (2007). New terpenoids from two Indonesian marine sponges. Nat. Prod. Res..

[3] Ebada S.S., Linh M.H., Longeon A., de Voogd N.J., Durieu E., Meijer L., Bourguet-Kondracki M., Singab A.N.B., Müller W.E.G., Proksch P. (2015). Dispacamide E and other bioactive bromopyrrole alkaloids from two Indonesian marine sponges of the genus *Stylissa*. Nat. Prod. Res..

[4] Ibrahim S.R.M., Mohamed G.A., Fouad M.A., El-Khayat E.S., Proksch P. (2009). Iotrochotamides I and II: New ceramides from the Indonesian sponge *Iotrochota purpurea*. Nat. Prod. Res..

[5] Zhang H., Loveridge S.T., Tenney K., Crews P. (2015). A new 3-alkylpyridine alkaloid from the marine sponge *Haliclona* sp. and its cytotoxic activity. Nat. Prod. Res..

[6] Gallimore W.A., Cabral C., Kelly M., Scheuer P.J. (2008). A novel D-ring unsaturated A-nor sterol from the Indonesian sponge, *Axinella carteri* Dendy. Nat. Prod. Res..

[7] Balansa W., Trianto A., de Voogd N.J., Tanaka J. (2017). A polyacetylenic alcohol from a sponge *Callyspongia* sp.. Nat. Prod. Commun..

[8] Ito T., Nguyen H.M., Win N.N., Vo H.Q., Nguyen H.T., Morita H. (2018). Three new sesquiterpene aminoquinones from a Vietnamese *Spongia* sp. and their biological activities. J. Nat. Med..

[9] Alvarez-Manzaneda E., Chahboun R., Cabrera E., Alvarez E., Haidour A., Ramos J.M., Alvarez-Manzaneda R., Romera J.L., Escobar M.A., Messouri I. (2008). A new synthetic strategy towards bioactive merosesquiterpenoids. Synthesis.

[10] Garai S., Mehta G. (2014). Total synthesis of bioactive drimane–epoxyquinol hybrid natural products: Macrophorin A, 40-oxomacrophorin A, and 10-epi-craterellin A. Tetrahedron Lett..

[11] Li J., Gu B.B., Sun F., Xu J.R., Jiao W.H., Yu H.B., Han B.N., Yang F., Zhang X.C., Lin H.W. (2017). Sesquiterpene quinones/hydroquinone from the marine sponge *Spongia pertusa* Esper. J. Nat. Prod..

[12] De Rosa S., De Giulio A., Iodice C. (1994). Biological effects of prenylated hydroquinones: Structure activity relationship studies in antimicrobial, brine shrimp and fish lethality assays. J. Nat. Prod..

[13] Sarin P.S., Sun D., Thornton A., Muller W.E.G. (1987). Inhibition of replication of the etiologic agent of acquired immune deficiency syndrome (human T-lymphotropic retrovirus/lymphadenopathy-associated virus) by avarol and avarone. J. Nat. Cancer. Inst..

[14] Loya S., Tal R., Kashman Y., Hizi A. (1990). Illimaquinone, a selective inhibitor of the RNase H activity of human immunodeficiency virus type-1 reverse transcriptase. Antimicrob. Agents Chemother..

[15] Rangel H.R., Dagger F., Compagnone R.S. (1997). Antiproliferative effect of Illimaquinone on *Leishmania mexicana*. Cell Biol. Int..

[16] Du L., Zhou Y.D., Nagle D.G. (2013). Inducers of hypoxic response: Marine sesquiterpene quinones activate HIF-1. J. Nat. Prod..

[17] Arai M., Kawchi T., Sato H., Setiawan A., Kobayashi M. (2014). Marine spongian sesquiterpene phenols, dictyoceratine-C and smenospongidiol, display hypoxia-selective growth inhibition against cancer cells. Bioorg. Med. Chem. Lett..

[18] Kong D., Aoki S., Sowa Y., Sakai T., Kobayashi M. (2008). Smenospongine, a sesquiterpene aminoquinone from a marine sponge, induces G1 arrest or apoptosis in different leukemia cells. Mar. Drugs.

[19] Gordazile M. (2010). Cytotoxic terpene quinones from marine sponges. Mar. Drugs.

[20] Luibrand R.T., Erdman T.R., Volmer J.J., Scheuer P.J., Finer J., Clardy J. (1979). Illimaquinone, a sesquiterpenoid quinone from a marine sponge. Tetrahedron.

[21] Kondracki M.L., Guyot M. (1987). Smenospongine: A cytotoxic and antimicrobial aminoquinone isolated from *Smenospongia* sp.. Tetrahedron Lett..

[22] Kushlan D.M., Faulkner D.J., Parkanyi L., Clardy J. (1989). Metabolites of the Palauan sponge *Dactylospongia* sp.. Tetrahedron.

[23] Takahashi Y., Kubota T., Ito J.P., Mikami Y., Fromont J., Kobayashi J. (2008). Nakijiquinones G–I. New sesquiterpenoid quinones from marine sponge. Bioorg. Med. Chem..

[24] Salmoun M., Devijver C., Daloze D., Braekman J.C., Gomez R., de Kluijver M., Van Soest R.W.M. (2000). New sesquiterpene/quinones from two sponges of the genus *Hyrtios*. J. Nat. Prod..

[25] Vargas S., Schuster A., Sacher K., Büttner G., Schätzle S., Läuchli B., Hall K., Hooper J.N.A., Erpenbeck D., Wörheide G. (2012). Barcoding sponges: An overview based on comprehensive sampling. PLoS ONE.

[26] Chombard C., Boury-Esnault N., Tillier S. (1998). Reassessment of homology of morphological characters in tetractinellid sponges based on molecular data. Syst. Biol..

