# Supplementary material for: A New Sesquiterpenoid Aminoquinone from an Indonesian Marine Sponge
Source: Mar Drugs. 2019 Mar 8;17(3):158. doi: 10.3390/md17030158 (PMC6470531; doi:10.3390/md17030158)
Supplement: Supplementary file 1 [file marinedrugs-17-00158-s001.pdf]

marine drugs

Supplementary Materials

A New Sesquiterpenoid Aminoquinone from an Indonesian Marine Sponge

Walter Balansa [1,2], Ute Mettal [1,3], Zerlina G. Wuisan [1,3], Anuchit Plubrukarn [4], Frans G. Ijong [2,5], Yang Liu [1,3,*] and Till F. Schäberle [1,3,6,*]

1 Institute for Insect Biotechnology, Justus-Liebig-University of Giessen, 35392 Giessen, Germany; walterbalansa1@gmail.com (W.B.); Ute.Mettal@chemie.uni-giessen.de (U.M.); Zerlina.G.Wuisan@bio.uni-giessen.de (Z.G.W)
2 Department of Fisheries and Marine Science, Nusa Utara Polytechnic, 95812 Tahuna, North Sulawesi, Indonesia; ijongfrans@yahoo.com
3 Department of Bioresources of the Fraunhofer Institute for Molecular Biology and Applied Ecology, 35394 Giessen, Germany
4 Department of Pharmacognosy and Pharmaceutical Botany, Faculty of Pharmaceutical Sciences, Prince of Songkla University, 90110 Songkhla, Thailand; anuchit.pl@psu.ac.th
5 Faculty of Fisheries and Marine Science, Sam Ratulangi University, 95115 Manado, Indonesia
6 German Center for Infection Research (DZIF), Partner Site Giessen-Marburg-Langen, 35392 Giessen, Germany
* Correspondence: Liu.Yang@agrar.uni-giessen.de (Y.L.); Till.F.Schaeberle@agrar.uni-giessen.de (T.F.S.); Tel.: +49-641-99-37140 (T.F.S.)

Contents

Figure S1. ^{1}H NMR spectrum of compound **1** (600 MHz, CD_3OD, δ in ppm).

Figure S2. ^{13}C NMR spectrum of compound **1** (150 MHz, CD_3OD, δ in ppm).

Figure S3. COSY spectrum of compound **1** (CD_3OD, δ in ppm).

Figure S4. HSQC spectrum of compound **1** (CD_3OD, δ in ppm**).**

Figure S5. HMBC spectrum of compound **1** (CD_3OD, δ in ppm).

Figure S6. NOESY spectrum of compound **1** (CD_3OD, δ in ppm).

Figure S7. LC-HRESIMS of compound **1**.

Figure S8. UV spectra of compounds **1**–**4**.

Figure S9. Diagrams for MIC determination of compounds **2**–**4**.

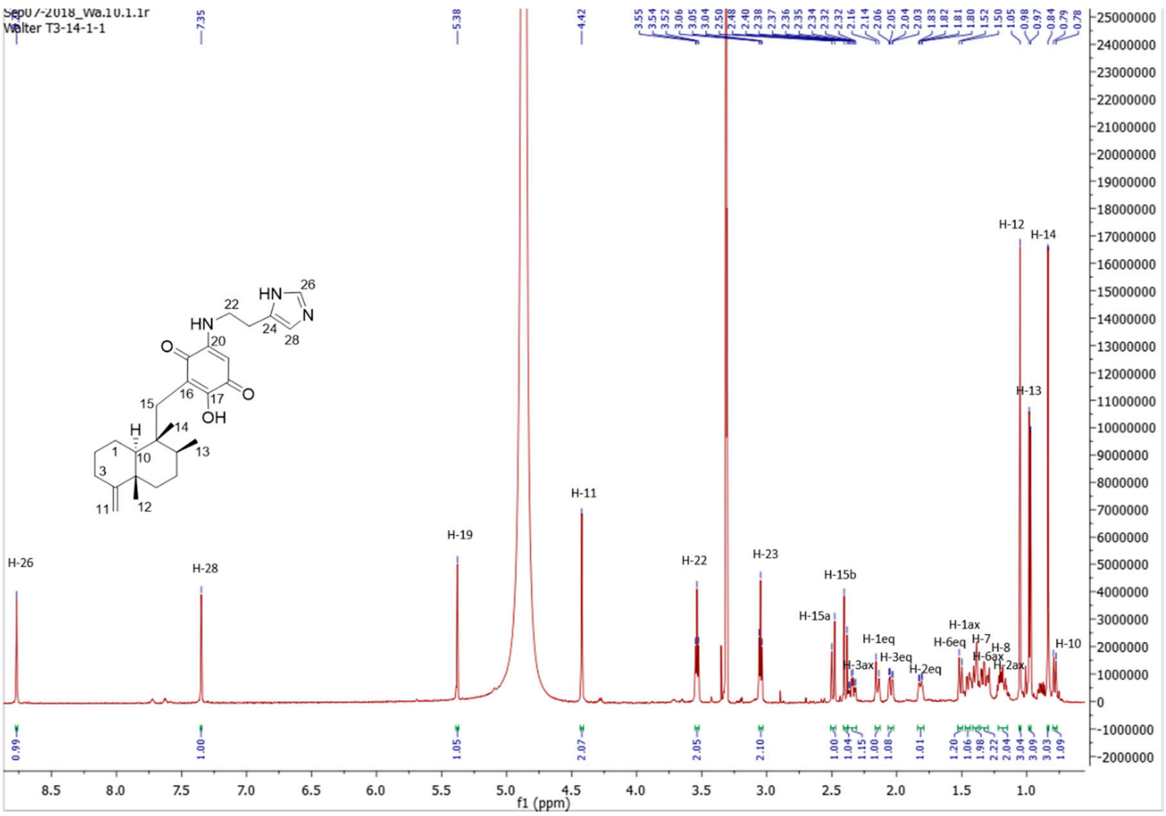

Figure S1. ^{1}H NMR spectrum of compound **1** (600 MHz, CD_3OD, δ in ppm).

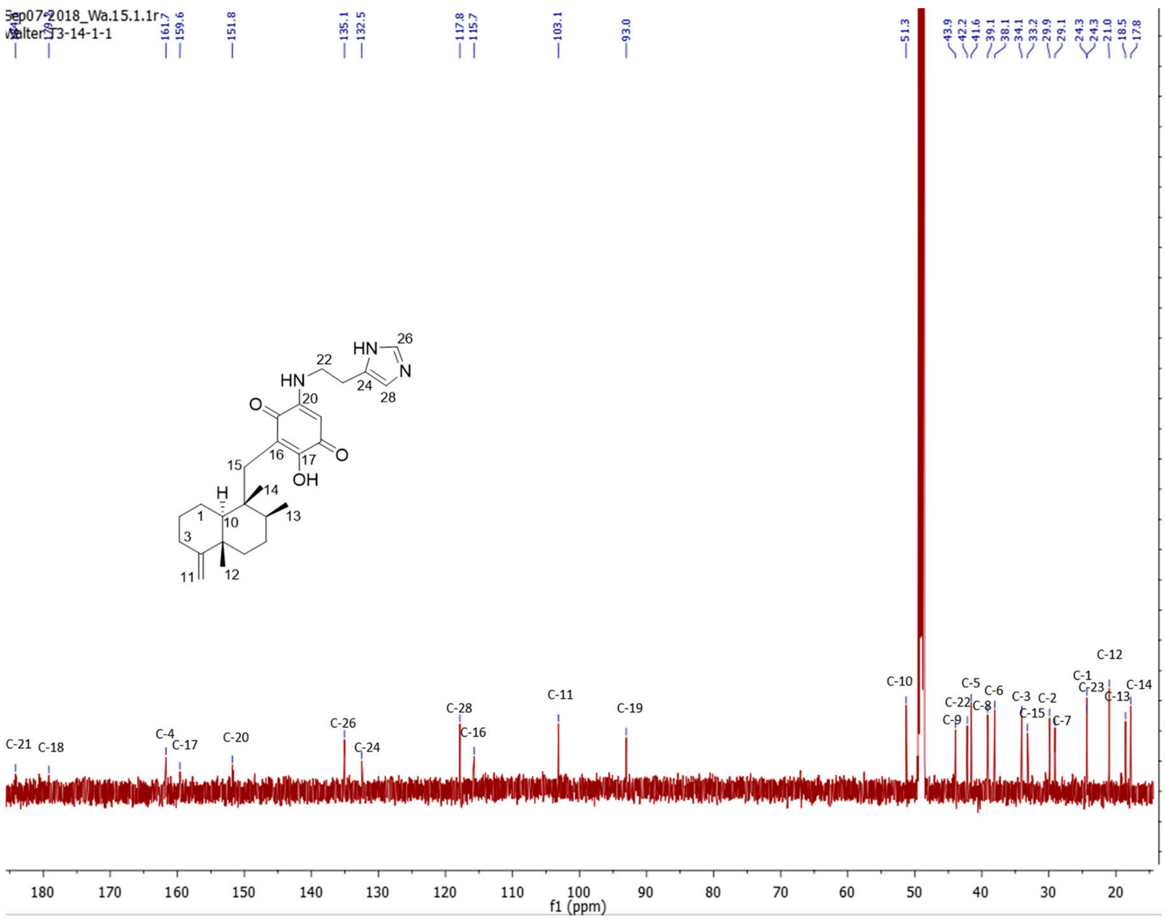

Figure S2. ^{13}C NMR spectrum of compound **1** (150 MHz, CD_3OD, δ in ppm).

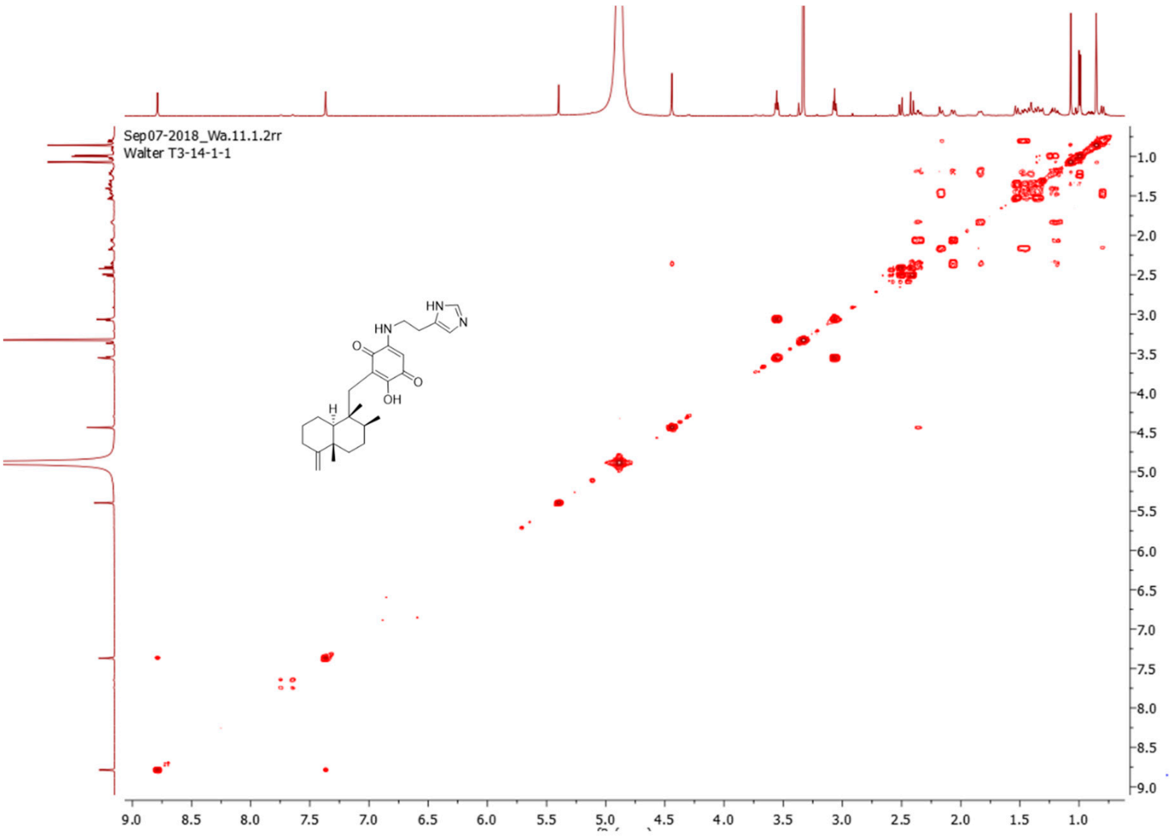

Figure S3. COSY spectrum of compound **1** (CD_3OD, δ in ppm).

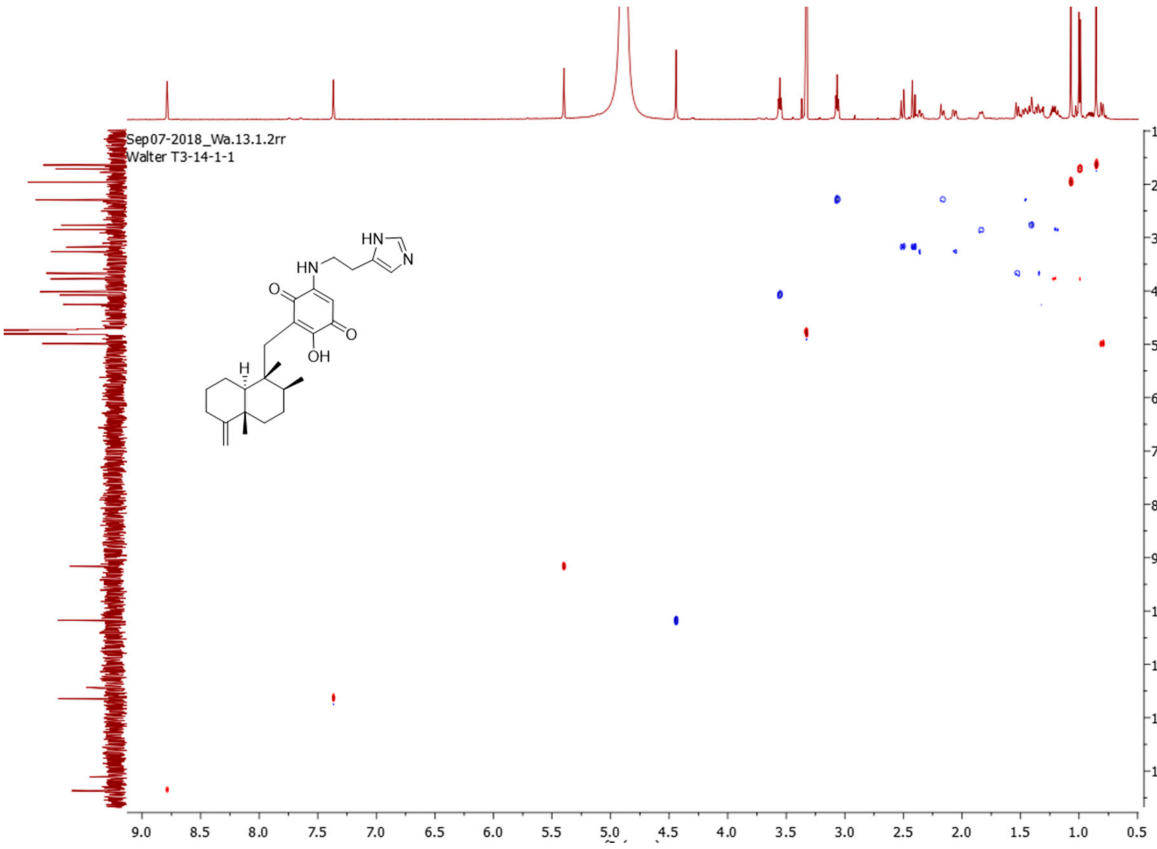

Figure S4. HSQC spectrum of compound **1** (CD_3OD, δ in ppm).

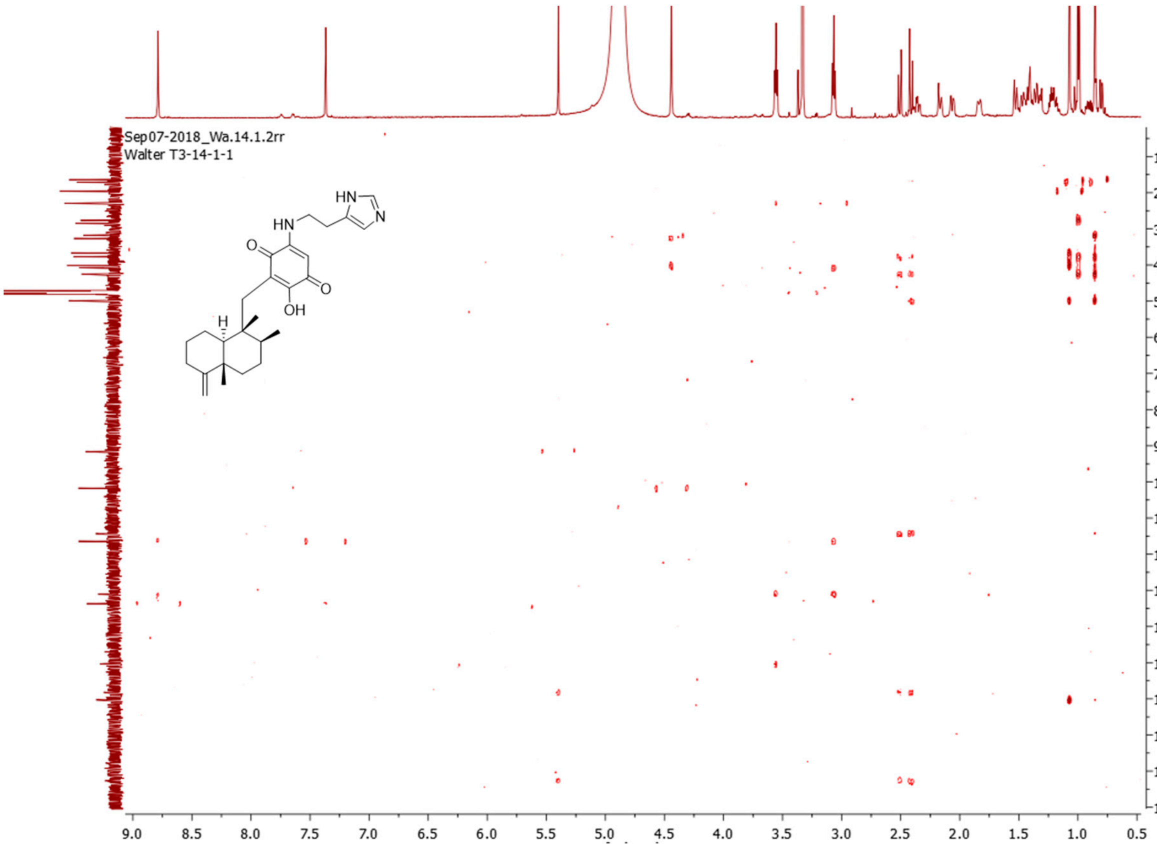

Figure S5. HMBC spectrum of compound **1** (CD_3OD, δ in ppm).

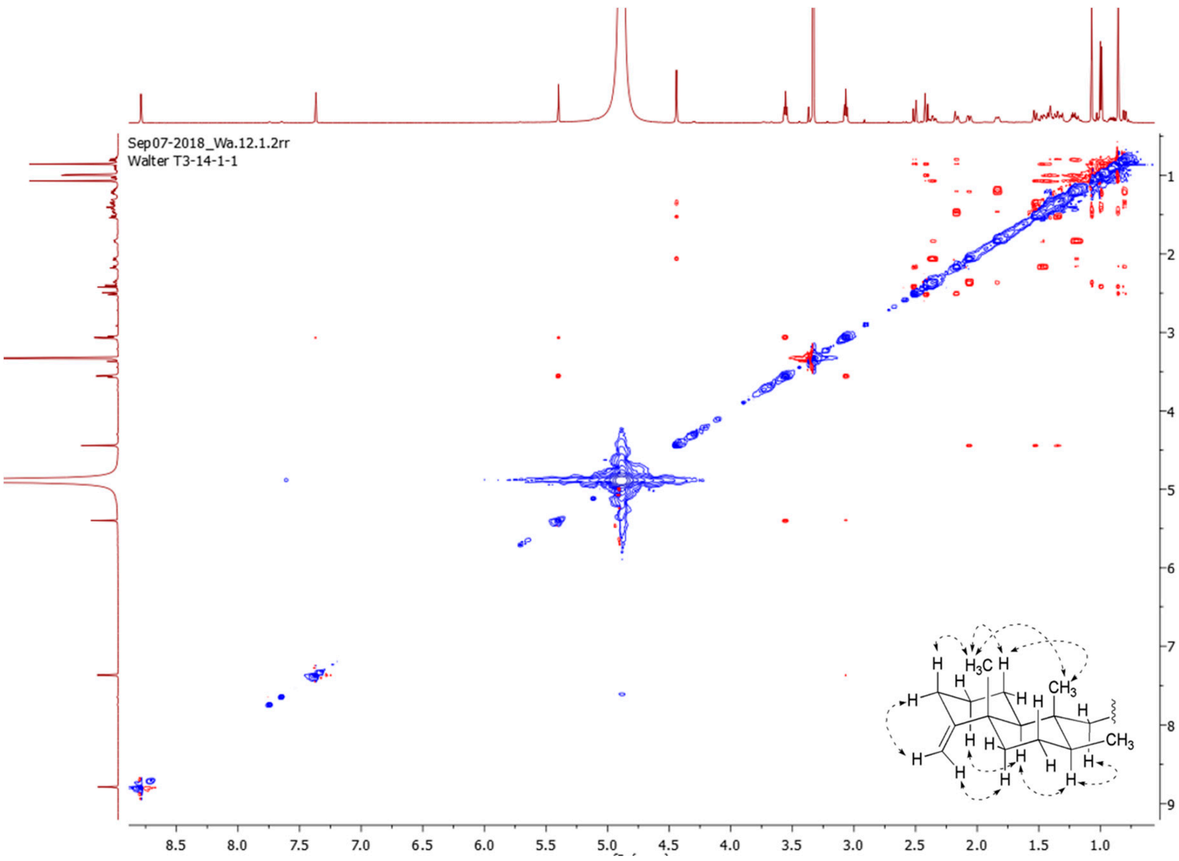

Figure S6. NOESY spectrum of compound **1** (CD_3OD, δ in ppm).

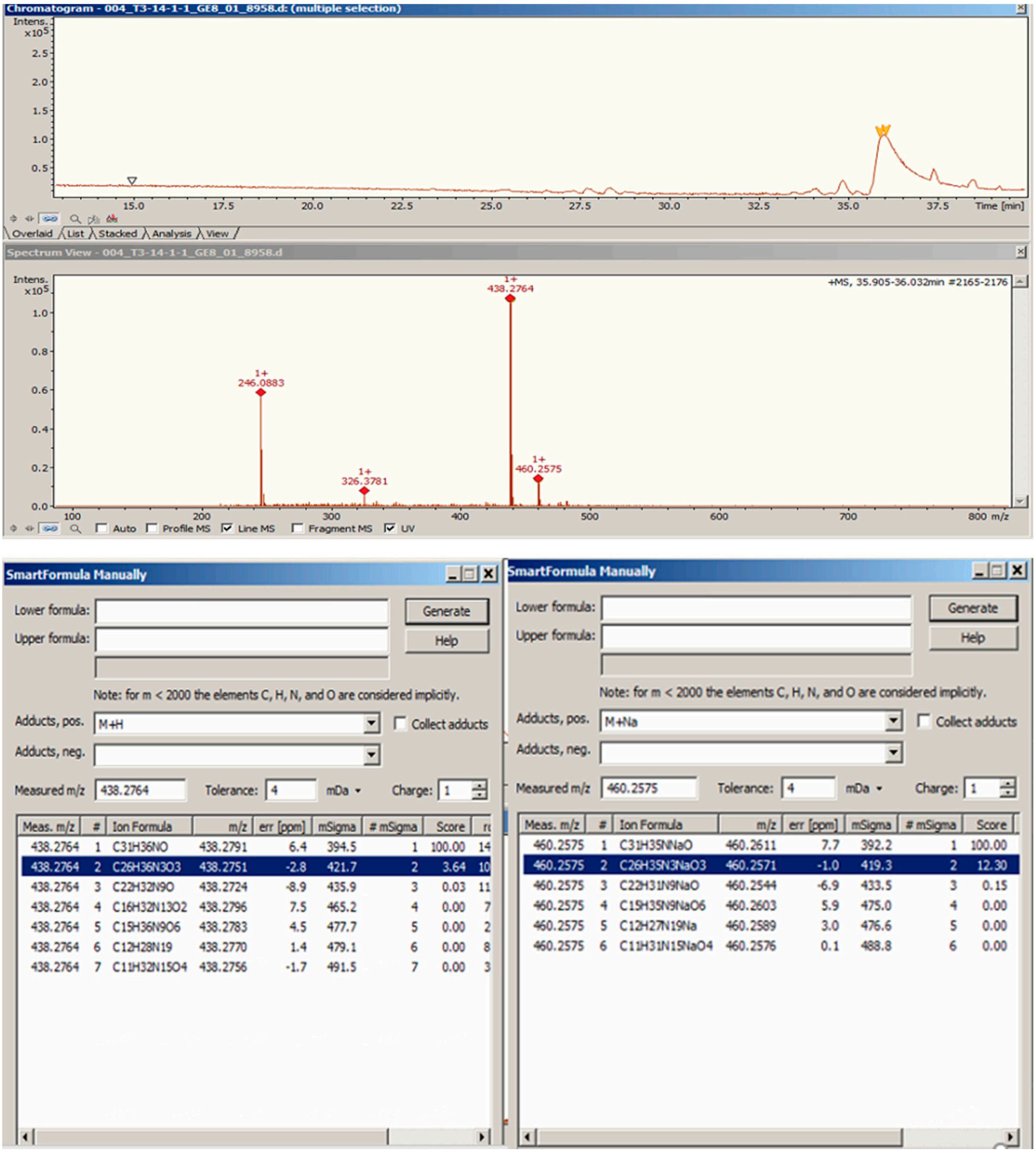

Figure S7. LC-HRESIMS of compound **1.**

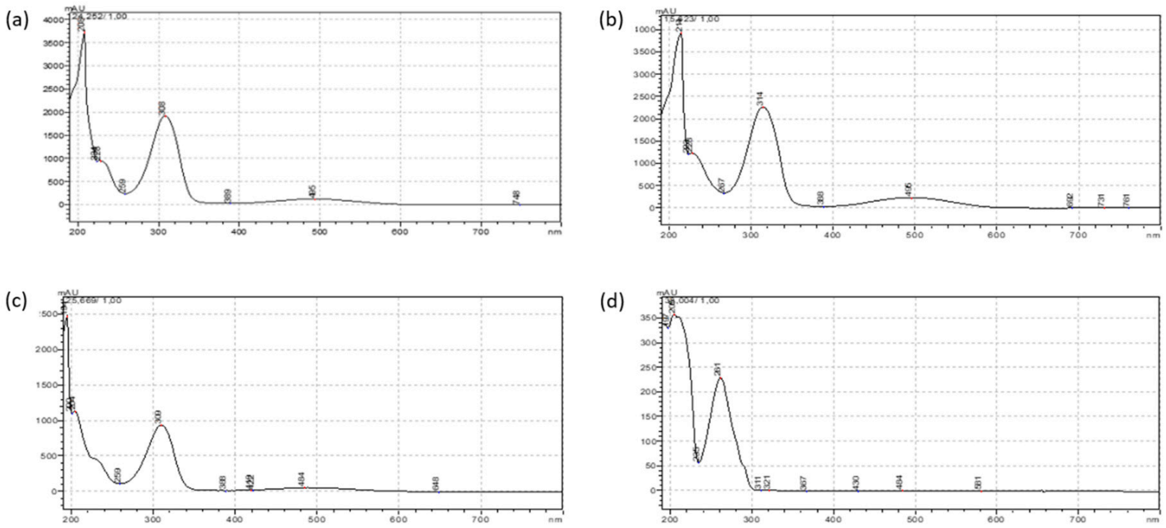

Figure S8. UV spectra of compounds **1–4**. (**a**) UV absorption of compound **1**; (**b**) UV absorption of compound **2**; (**c**) UV absorption of compound **3**; (**d**) UV absorption of compound **4**.

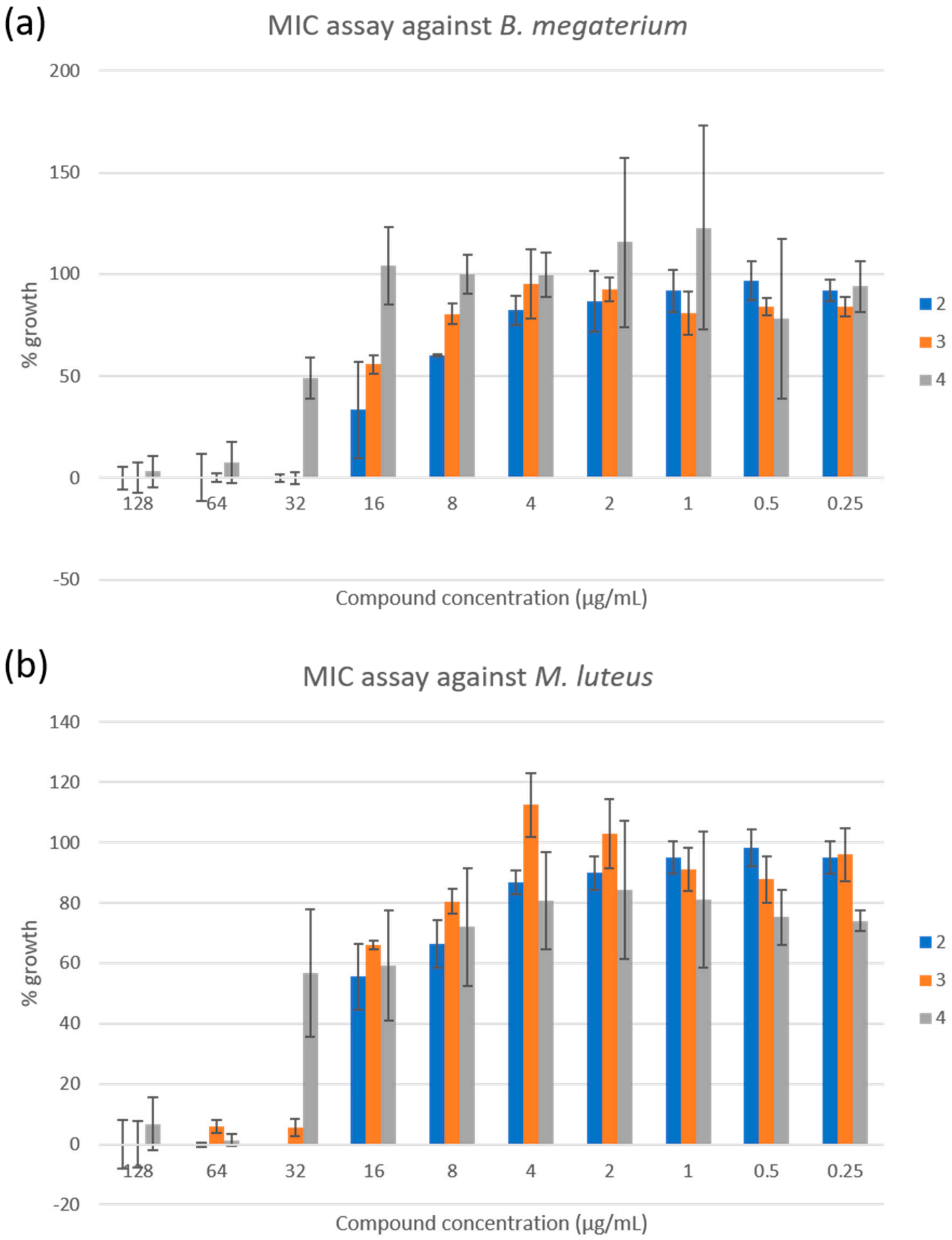

Figure S9. Diagram for MIC determination of compounds **2–4.** (**a**) MIC diagram of compound **2–4** against *B. megaterium*; (**b**) MIC diagram of compound **2–4** against *M. luteus.*
